# COVID-19 Pandemic on smoking behavior changes among African American Smokers Eligible for LDCT Screening

**DOI:** 10.1093/geroni/igab046.2736

**Published:** 2021-12-17

**Authors:** Tung-Sung Tseng, Yu Hsiang Kao, Mirandy Li

**Affiliations:** 1 LSUHSC, New Orleans, Louisiana, United States; 2 LSUHSc, New Orlans, Louisiana, United States

## Abstract

Smoking has been observed to associate with an elevated severity of disease and risk of mortality among people with COVID-19. Additionally, African American smokers have higher rates of mortality from lung cancer than other racial/ethnic groups. Low dose computed tomography (LDCT) screening can detect lung cancer early to decrease lung cancer-specific mortality for current smokers but remains under-utilized among these population. However, we know little about the effect of COVID-19 pandemic on smoking behavior changes among African American smokers who qualify for LDCT screening. This study recruited 60 African American daily smokers seen in primary care clinics, who qualified to receive LDCT screening in a New Orleans, LA hospital. A total of 22 participants (36.7%) completed anonymous cross-sectional survey that collected demographic, disease history, tobacco use, and smoking cessation behaviors during the period of COVID-19 pandemic via phone interview. The majority were older (61.2 [SD=4.7]), female (77.3%), earned annual income less than $20,000 (100.0%), had Medicaid (63.6%), overweight/obesity (72.7%), planned to quit smoking within 6 months (52.4%), and would consider taking LDCT screening after COIVD-19 pandemic (95.2%). Half of smokers reported they have been diagnosed hypertension (47.6%), diabetes (52.4%), and arthritis (57.1%). Regarding health behavior changes, 42.9% smokers reported they smoked more, felt more stress (42.9%) and anxiety (33.4%) after COVID-19 outbreak. Smoking cessation programs may focus on this high-risk minority population in the post COVID-19 pandemic to help them decrease cigarette smoking and enhance their motivation to quit smoking.

